# Engaging stakeholders in implementation research: lessons from the Future Health Systems Research Programme experience

**DOI:** 10.1186/s12961-017-0269-6

**Published:** 2017-12-28

**Authors:** David H. Peters, Abbas Bhuiya, Abdul Ghaffar

**Affiliations:** 1 Department of International Health, Johns Hopkins Bloomberg School of Public Health, Johns Hopkins University, 615 North Wolfe Street, Baltimore, MD 21205 United States of America; 2 Alliance for Health Policy and Systems Research, World Health Organization, Geneva, Switzerland

Implementation research and the engagement of stakeholders in such research have become increasingly prominent in finding ways to design, conduct, expand and sustain effective and equitable health policies, programmes and related interventions [1]. How to bring together key sets of health systems stakeholders, including affected communities, health workers, health system managers, health policy-makers and researchers, as well as non-state and non-health sector actors, is a critical challenge. Stakeholder engagement plays important roles across intersecting research, policy and management processes, from selecting and defining the most relevant research questions to address policy and management concerns, to designing and conducting research, learning from and applying evidence, making changes to policy and programmes, and holding each other accountable [2]. The articles in this supplement examine some of the tools and approaches used to facilitate stakeholder engagement in implementation research, and describe learning from the experience of the Future Health Systems (FHS) Research Programme Consortium. Over the past decade, the FHS Consortium, comprised of teams from Afghanistan, Bangladesh, China, India and Uganda, have worked closely with the people and organisations leading the transformation of health systems in each of their own countries. They have pursued approaches that allow key actors to ‘learn by doing’. In doing so, they find how implementation research can be usefully employed by providers, beneficiaries, officials and key local actors to improve the delivery of health services, particularly for poor and marginalised populations [3].

The articles in this series demonstrate how teams are able to reflect on learning processes that occurred through interactions between researchers and other stakeholders. They have taken place over many years, allowing the teams to compare experiences across different countries, tools and stakeholder groups, as well as a range of conceptual models. Paina et al. [4] outline how teams in Bangladesh, India and Uganda used theories of change to facilitate iterative interactions with different stakeholders and the design and implementation of interventions. Typically, theories of change are used by project planners or researchers to clarify the design or evaluation logic of a project. In the cases described, the theories of change and their revision processes provided useful platforms for planning, communication and learning for those implementing the programmes and with intended beneficiaries. In doing so, the theories of change helped to highlight accountabilities of key actors.

Ekirapa-Kiracho et al. [5] discuss two participatory methods for engaging with stakeholders, namely Participatory Social Network Analysis and Participatory Impact Pathways Analysis (PIPA) in India and Uganda, and derive lessons about when and how to apply these tools. They found that, whereas both methods helped to identify stakeholders and provide a deeper understanding of the type of networks and dynamics within the network, PIPA had a higher potential for promoting collaboration between the stakeholders, and to help provide an evaluation of the programme from the perspective of the communities affected. Kananura et al. [6] further examine how PIPA and other active monitoring and evaluation techniques were able to bring together researchers, village health teams and their supervisors at sub-county and district levels in Uganda. They were able to identify critical problems and identify feasible solutions using a number of techniques to share data and reflection. As teams implemented solutions, they continued to carry on with further problem-solving cycles, gaining confidence in their ability to solve problems.

The Uganda team also explored the influence of Participatory Action Research (PAR) on strengthening management capacity in Eastern Uganda [7]. A key characteristic of PAR is that it enables participants to do their own research as a basis for actions they will take. In this case, the Uganda team is using PAR to focus on implementation issues in the healthcare system. Their findings indicate that a PAR approach enhanced health managers’ capacity to collaborate with others, be creative, attain goals and review progress. The enabling factors included the expanded interaction spaces, encouragement of flexibility, empowerment of local managers and the promotion of reflection and accountability. Tension and conflict across different management functions was apparent, particularly to balance collaboration and control over some processes. These tensions meant that managers needed to learn to simultaneously draw upon and use different competencies, which the authors demonstrate through use of a Competing Values Framework. This shows how building management capacity is a complex process, and can be enhanced by finding ways to bring different perspectives and stakeholders together, in part to overcome conflicts that emerge.

Bennett et al. [8] draw upon three studies conducted through FHS in Afghanistan, Bangladesh and Uganda. All three cases involved complex interactions between the research teams and other stakeholders, among different stakeholders, and between stakeholders and the intervention. The research planned by the research teams focused primarily on feasibility and effectiveness, however, in practice, the research teams also had critical insights into other factors such as sustainability, acceptability, cost-effectiveness and appropriateness. In each case, in addition to stakeholder analyses, other project management tools were used to engage stakeholders in addressing implementation issues, which served to help the projects succeed and complement the primary research.

The Roundtable discussion provides interesting insights from the perspective of health system policy-makers and managers in three countries [9]. All highlight the importance of research to inform decisions, and identify a number of successful ways in which they have worked, as well as some of the remaining challenges. All three point to the need for continued capacity-building to produce and use research, with the policy-maker from India noting that researchers still have a long way to go in presenting research in ways that enable policy-makers to use the research findings to make informed decisions. The health systems managers from Uganda and Afghanistan highlight the gains made by expanding partnerships with community members and researchers. Whereas their experience has raised their own expectations, they also feel that they are now able to use data in their daily work and take on new challenges, and better meet the needs of their communities.

Each of the articles embody a collaborative approach to ‘learning by doing’, which is particularly appropriate for addressing implementation issues. Implementation research is providing significant insights into how to enhance the acceptability, fidelity and reach of health interventions, particularly for disadvantaged people. However, in order to take advantage of the capabilities of stakeholders and be able to address continuously emerging challenges, it seems that considerable flexibility is needed in both how research is conducted, as well as how interventions are implemented based on this research. In other words, to continue to learn and do.
